# UK and Australian University Students’ Perceptions of the Nature of Sexual Assault and Intervening Behavior

**DOI:** 10.1177/08862605231212171

**Published:** 2023-11-14

**Authors:** Danielle Labhardt, Sarah Brown, Emma Holdsworth, Nadine McKillop, Douglas James Howat, Christian Jones

**Affiliations:** 1 Manchester Metropolitan University, UK; 2 University of the Sunshine Coast, Sippy Downs, QLD, Australia; 3 Coventry University, UK

**Keywords:** sexual assault, bystander intervention, prevention, consent, qualitative, student perceptions

## Abstract

Sexual assault is a global problem, with the risk highest among university students. Bystander intervention preventing sexual assaults has primarily been researched using quantitative methods to understand what factors influence it. However, both sexual assault and bystander intervention are complex with many subtle and overlapping issues that, when analyzed qualitatively, can offer new insights. The current study aimed to explore and develop a nuanced and comprehensive understanding of students’ perceptions of sexual assault and bystander intervention across two universities, one in the United Kingdom and one in Australia. Thirty-nine university students (19 in the United Kingdom; 20 in Australia) took part in one-to-one semistructured interviews. Using inductive thematic analysis, two overarching themes were identified: (a) navigating the complex dynamics of sexual assault; and (b) decisions to intervene or not to intervene. Findings suggest that the complexity and ambiguity around sexual assault can forestall bystander intervention. As such, increasing education, awareness, and discussions around sexual assault and bystander intervention is vital to increase awareness of the problem and mobilize action from bystanders to prevent sexual assault.

## Introduction

Sexual assault is an ongoing, global problem (Abrahams et al., 2014; Kimble et al., 2008; Martin et al., 2011; Senthilingam, 2017), especially among university students (Ministry of Justice, 2013; National Union of Students, 2010). Between August 2009 and March 2010, one in seven students at UK universities were sexually assaulted (National Union of Students, 2010) and 75% reported an “unwanted sexual experience” (National Union of Students, 2019). In Australia, estimates are that between one in four (National Union of Students, 2015) and 1 in 10 (Australian Human Rights Commission, 2017) students are sexually assaulted. More recently, one in three Australian university students reported sexual assault at some point during their lives and of those, 1 in 20 experienced sexual assault in a university context (Heywood et al., 2022).

To address this issue, universities have implemented consent awareness programs and/or bystander intervention programs (Fenton et al., 2016; Universities UK, 2016). Evidence suggests that the Green Dot campaign (Coker et al., 2015) was successful in decreasing sexual victimization (Fenton et al., 2016). However, a recent review concluded that while there were positive short-term effects on intervention behavior, there was still inconsistency between attitudes/perceptions and bystander behavior (Kettrey et al., 2019), highlighting the complex and multidimensional nature of attitudes and bystander intervention (McMahon, 2015). Moreover, researchers have argued that qualitative methods are needed to better understand perceptions of sexual assault, bystander intervention, and the underlying constructs that affect the likelihood of intervening (Banyard, 2015; McMahon, 2015), providing the impetus for this study.

### Defining the Problem of Sexual Assault

In the United Kingdom, sexual assault is defined as one person touching another in a sexual manner without consent (GOV.UK, 2004). Similarly, in Australia, sexual assault is defined as “acts, or intent of acts, of a sexual nature against another person, which are nonconsensual or where consent is proscribed” (Australian Bureau of Statistics, 2008, p. 23). Although wording varies, fundamental to definitions are the lack of consent and sexual motivation.

Prevention practice originally adopted the “no means no” approach; however, in recent years, the focus has shifted toward affirmative consent, focusing on “yes means yes” (Beres, 2018; Dougherty, 2015). Affirmative consent is active and ongoing; “nonconsent must be assumed until consent is actively communicated” (Muehlenhard et al., 2016, p. 464). Affirmative consent can be conceptualized via an “internal state of willingness,” not directly observable, explicitly by verbally consenting to sexual activity, or through body language (Muehlenhard et al., 2016, p. 462). However, challenges remain regarding affirmative consent (Jozkowski et al., 2014; Muehlenhard et al., 2016). Explicit verbal consent could reduce ambiguity, but verbal consent remains rare; in fact, the voice is more often used to indicate nonconsent (Muehlenhard et al., 2016). Evidence suggests that university students rely on nonverbal methods to communicate consent, such as touching their partner, or not resisting advances (Beres et al., 2004; Jozkowski & Wiersma, 2015; Wiederman, 2005) furthering the possibility for misinterpretation and miscommunication (Fernet et al., 2021; Muehlenhard et al., 2016; Wilson & Miller, 2016).

Consent Matters is a well-known awareness program used within the United Kingdom and Australia that facilitates positive change and instills knowledge and confidence regarding sexual consent (Epigeum, 2019a). To the authors’ knowledge, no formal evaluations of the program have been conducted. However, testimonials suggest the program has a positive impact (Epigeum, 2019a) and can be beneficial in raising awareness. Nevertheless, other influences such as traditional gender norms (men as initiators and women as gatekeepers) and contextual factors, such as alcohol (Brady et al., 2018; Jozkowski et al., 2017; Jozkowski & Wiersma, 2015; Muehlenhard et al., 2016; Ortiz, 2019), further contribute to the complexities of sexual consent. Understanding how university students understand consent and its complexities is important, as they can influence whether they perceive a need to intervene as a bystander.

### Bystander Intervention

In addition to consent awareness programs, some universities have also implemented bystander intervention programs as a way of preventing sexual assaults (e.g., the bystander initiative toolkit; Fenton et al., 2016). The Consent Matters training version 2 has also integrated bystander intervention within its program for the purpose of improving sexual assault prevention (Epigeum, 2019b). This integrated approach enables students and staff to develop knowledge around consent and prevention, build resiliency skills, and develop safe strategies for intervening in a sexual assault (Fenton et al., 2016). Fenton et al.’s review demonstrates that bystander intervention appears to be effective, reporting a decrease in prevalence from 20% to 11%.

Latané and Darley’s (1970) five-step bystander intervention model has been employed to understand the factors that influence responses to a potential assault: witnesses must (a) notice the event; (b) perceive it as an emergency; (c) take responsibility; (d) identify how to intervene; and (e) intervene. A plethora of quantitative research has focused on sexual assault and the effect different factors have on intervention by witnesses to a sexual assault (see Labhardt et al., 2017 for a review). Much of this research is USA-based, with only limited studies in the United Kingdom (e.g., Camp et al., 2018) or Australia (e.g., Kania & Cale, 2018). However, the objectives of the research remain the same: to identify how different factors influence the likelihood of bystander intervention.

Findings demonstrate that some factors appear to be consistent in predicting bystander intervention, while others remain inconsistent. For instance, the seriousness of the situation can affect the likelihood of bystander intervention (Baillie et al., 2022; Fischer et al., 2006; Pugh et al., 2016; Yule & Grych, 2017). If it is not clear whether or not consent was given (Brady & Lowe, 2020; Brady et al., 2018), or it is not clear what the relationship between the people are (Banyard & Moynihan, 2011; Burn, 2009), a potential bystander may not see the situation as serious enough to intervene. Research has consistently demonstrated that the likelihood of intervention decreases as the number of witnesses increases. However, when a bystander perceives their peers to be supportive, the likelihood of intervention increases (Banyard & Moynihan, 2011; Banyard et al., 2014; Brown et al., 2014; Katz et al., 2015).

These findings illustrate the complexity of the factors that may influence and inhibit intervention. Burn (2009) argued, in line with Latané and Darley’s (1970) model, that failing to notice an event (focused on one’s own behavior, e.g., partying), the ambiguity of the situation (alcohol impacting on perceived seriousness), believing it is not the bystander’s responsibility to intervene (diffusion of responsibility), and/or not knowing how to intervene (skills deficit) can prevent a bystander from intervening. Furthermore, Leone et al. (2018) argued that alcohol decreases the likelihood of intervention as intoxicated potential bystanders might not notice the event or accurately interpret cues.

### The Present Study

Research has shown that many factors associated with the perceptions of the event (e.g., its seriousness, type of relationship) and related to the bystander (e.g., alcohol intoxication) influence whether bystanders identify a situation as needing intervention. Perceptions are further complicated by the tendency to use nonverbal cues to indicate consent that are difficult to interpret. While interventions aiming to increase bystander intervention have some positive outcomes, there remains inconsistency between perceptions and behavior, which warrant further investigation. Research to date has been predominately quantitative, which does not provide a good understanding of the complex and overlapping factors that influence bystander intervention, or the subtle differences in cues that influence perceptions that guide behaviors. Given the higher risk of sexual assault among university students, it is important to qualitatively explore their perceptions of sexual assault, what it means to be a bystander, and the factors that influence intervention. Taking a qualitative approach complements existing findings by offering a more nuanced understanding of the complex dynamics that support (or hinder) intervention efforts; to date, limited qualitative studies have been conducted, even less in the United Kingdom and Australia. The qualitative exploration of university students’ perceptions of sexual assault and bystander intervention in these two countries will help to inform preventative efforts at a local and global scale.

Two research questions guided this study. First, how do university students at a UK and an Australian university understand and perceive sexual assault and consent, and second what are university students’ perceptions and understanding of being a bystander and the factors that influence bystander intervention.

## Methodology

### Participants

In accordance with Terry et al. (2017) guidelines, participants were 19 students from a UK university and 20 students from an Australian university. UK participants were not provided with an incentive for participation. However, participants from the Australian university were provided with an AUD$20 monetary incentive, which was not contingent on completing the study. Pseudonyms were allocated to maintain confidentiality. Demographic details are provided in Table 1.

**Table 1.** Sample Demographics.

	Cross-Cultural Sample *N* = 39	
Demographics	UK (*n* = 19)	Australia (*n* = 20)
Gender		
Male	31.6% (*n* = 6)	35.0% (*n* = 7)
Female	68.4% (*n* = 13)	65.0% (*n* = 13)
Mean Age (SD) in years	20.32 (2.41)	30.75 (11.50)
Range in years	18–26	18–52
Relationship status		
Single	68.4% (*n* = 13)	60.0% (*n* = 12)
In a relationship	31.6% (*n* = 6)	40.0% (*n* = 8)

### Data Collection

**Figure 1.**
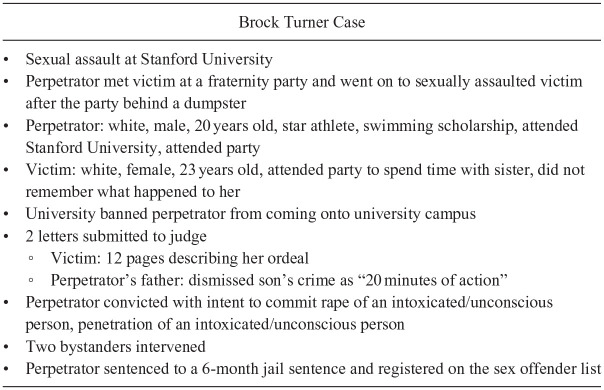
The Brock Turner Case Used in the Participant Interview Process.

To explore participants’ perceptions of sexual assault, semistructured one-to-one interviews were conducted. Semistructured interviews offer flexibility by adjusting and adapting questions based on responses (Fylan, 2005). As a prompt to discussion and to overcome potential hesitancy (Fylan, 2005), five information points associated with sexual assault and bystander intervention (see Table 2) and a case study on the Brock Turner case (see Figure 1) (Baker, 2016) were provided to participants. Participants were presented with an information point and then asked questions relating to the point (e.g., “what is your understanding of consent?” or “can you describe what a risky situation looks like?”). This approach facilitated conversation and allowed participants to reflect on what the points meant to them and how their own perceptions related to the awareness and knowledge of others. The Brock Turner case was used as it was a recent, highly publicized case, which people would likely be familiar with (Stack, 2016). Participants who were not familiar with the case were given further information about it such as who intervened, and how, to help generate discussion.

**Table 2.** Information Points Used in the Participant Interview Process.

Five Facts		
Information Point	United Kingdom	Australia
1	Sexual assault—when one person intentionally touches another in a sexual manner without consent (GOV.UK, 2004)	Sexual assault—acts, or intent of acts, of a sexual nature against another person, which are nonconsensual or where consent is proscribed (Australian Bureau of Statistics, 2008)
2	Approximately one in seven UK university students are sexually assaulted every year (National Union of Students, 2010)	Approximately one in four Australian university students are sexually assaulted every year (National Union of Students, 2015)*
3	Risk of victimization is highest among women aged 16–19, who are studying full-time, and who visit pubs or night clubs at least once a week (Ministry of Justice, 2013)	Risk of victimization is highest among women aged 18–24 (Australian Bureau of Statistics, 2012), who study at university, and who visit pubs or night clubs at least once a week (Diego et al., 2002; Ministry of Justice, 2013)
4	Approximately 2% of victims of less serious sexual assault (i.e., touching, molesting, or unwanted kissing) report to either the police or the institution; approximately 10% (report to police) and 4% (report to the institution) of victims report serious sexual assault (i.e., attempted or successful rape/penetration) (National Union of Students, 2010)	Approximately 5.5% (report to the institution) and 4.8% (report to the police) of victims report sexual assault (National Union of Students, 2015)
5	Approximately 33% of witnesses of a sexual assault intervene (Burn, 2009; Planty, 2002)	

* Note. Once data collection commenced and most participants had been presented with the 1 in 4 figures, the Australian Human Rights Commission released a report from 39 Australian universities reporting that 1 in 10 students are sexually assaulted (Australian Human Rights Commission, 2017). To maintain consistency, the one in four statistics was presented to remainder of participants.

### Procedure

Ethical approval was obtained independently from both universities. UK participants were recruited at the end of 2016, and Australian participants were recruited at the end of 2017. Participants were recruited using SONA (UK institution’s online participant pool recruitment method) and BlackBoard (Australia institution’s online forum used to communicate with students). They were provided with detailed information about the study and their rights to withdraw. Informed consent was obtained prior to interviews, which were conducted in a private location on each university campus by the lead author and lasted approximately 60 min.

Each audio-recorded interview progressed with discussion on each information point and the Brock Turner Case. Participants were fully debriefed and provided with contact information for available support. Participants were offered the opportunity to review their interview transcript prior to analysis, to ensure their responses were accurately represented. Six UK participants and seven Australian participants asked to verify their transcripts. Responses from each country were transcribed and analyzed separately, then examined together to identify similarities and differences.

### Data Analysis

The first author transcribed the interviews verbatim and the transcripts were checked by the third author. Using NVivo (QSRInternational, n.d.), an inductive thematic analysis was employed as it offers a flexible approach to reflect reality, while providing rich, detailed, and complex accounts of the data. Furthermore, the authors used a realist approach (Braun & Clarke, 2006; Terry et al., 2017) to allow the findings to be data driven and limit the influence of personal preconceptions of the topic under investigation. The first author completed the initial coding and developed the initial themes, which were reviewed independently by the third author. Themes were adjusted and finessed based on critical discussion to ensure codes, extracts, and themes accurately reflected the participant voice. Final themes and extracts were reviewed, refined, and agreed upon by all authors.

## Results

Two overarching themes with six subthemes were derived from the data and are discussed in turn. Table 3 provides an overview of the themes.

**Table 3.** Themes and Subthemes.

1. Navigating the Complex Dynamics of Sexual Assault a. Sexual assault is subjectively defined b. The shared understanding of consent c. Consent is not a “yes,” it is the absence of a “no”
2. Decisions to Intervene or Not to Intervene a. Variability in identifying the need to intervene b. Ambiguity of event negatively impacts bystander intervention c. Support positively influences bystander intervention

### Navigating the Complex Dynamics of Sexual Assault

This theme reflects how individuals navigate through the complexity of what is sexual assault. This complexity allows for subjective interpretation among participants. The subthemes are sexual assault is subjectively defined; the shared understanding of consent; and consent is not a “yes,” it is the absence of a “no.”

#### Sexual Assault is Subjectively Defined

There was a consensus among all participants regarding the definition of sexual assault (see Table 2). Initially, when considering the definition of sexual assault (point 1 of Table 2), participants perceived the definition as “true, where it’s assumed, the victim does not necessarily give consent.” (Hannah [F], Australia) and that “if you touch someone without their consent in a sexual manner, it is sexual assault” (Michael [M], UK). The lack of consent on the victim’s behalf is the key factor that is the difference between consensual and nonconsensual sexual encounters. However, when discussed further, most participants described the definition as vague and subject to individual interpretation.

it’s very vague. Some people wouldn’t um, consider certain things sexual assault, but others would. So, the same situation would just be perceived differently, I guess. [. . .] some people might not actually um, perceive what’s happening as assault and some people would, depending on the situation. Not everyone views it the same. Emily (F), UK

This ambiguity in belief and interpretation hinders identifying whether sexual assault may be occurring. People may rely on multiple methods of giving consent. Body language could be inherently difficult to interpret, possibly leading to the misperceptions or misunderstanding of the situation.

#### The Shared Understanding of Consent

Participants were clear and consistent in their understanding of what consent means. They reported that consent is about “mutual agreement from both sides” (Joshua; M; UK). This demonstrates that both parties need to be active and willing participants for consent to be present. However, participants in Australia provided more detailed responses, possibly due to a program on campus called Consent is Sexy; a Sexual Rights Awareness Campaign (Consent is Sexy, 2011) that promotes respect, consent, and talk about sexual relationships.

I think it is a mutual and respectful word or saying you give someone permission and you give it freely and with full understanding. It’s not coerced and it’s informed. You have an understanding of what it means when you give it or when you received it from someone. Megan (F), Australia

Responses talked to the respect two people should show one another in a sexual relationship. Regardless of the type of relationship and the sexual act, two people need to explicitly consent to that activity.

That it’s basically an agreement to participate or to engage in some sort of reciprocal participation, whether that be like what we’re saying, whether it be in a sexual activity, or enjoyment in sexual banter, or physical sensations. What you’re saying is I choose to partake in this with you. Megan (F), Australia

I don’t care how much you’ve led someone on or stuff like that. You can always say no at the end of it. And I think that’s really important [. . .] even if it’s been leading up to that and you still don’t want to, it’s just as much of a sexual assault. Olivia (F), UK

Consent is a continuous, ongoing process, which can be revoked at any stage; it is not a blanket agreement. For example, agreeing to kissing, does not automatically equate to consenting to sex. However, while participants agree consent is essential, how consent can be provided varied among participants.

#### Consent Is Not a “Yes,” It Is the Absence of a “No”

While consent may be fluid, methods of giving consent also vary, increasing the complexity of the problem. Consent could be given verbally or via body language. Some participants expressed the belief that verbal consent is explicit and removes ambiguity.

Verbal communication is definitely more easier to interpret. Jessica (F), Australia

It clearly demonstrates whether a person is willing to engage in sexual activities. However, some participants perceived verbal consent to be awkward and unnatural regardless of the relationship. For them, consent was not about verbally agreeing. Instead, the absence of a “no” was perceived as having consent. In these cases, the primary method of giving consent was based on body language and the natural progression of sexual activities.

It’s not like you ask. Yes. Do you consent to me touching you? [. . .] I think you can tell from body language and stuff. If someone didn’t want it, you would be able to tell. [. . .] But if they reciprocate your reactions or you know, then obviously that’s giving consent. Chloe (F), UK

You don’t just go up to your wife and say okay let’s go to bed and have sex or make love or something like that. Well you might do, but it’s not very romantic. So, the start of a sexual act is usually physical romance of a physical nature, whether they be holding, kissing, touching, feeling, right. Till one of the parties is uncomfortable and says no. That’s it and that can work from the very onset. Right from the very first movement. Lucas (M), Australia

Consent via body language relies on understanding the body cues of another individual. That intimate connection between two people responding and reacting to nonverbal consent is how participants interpret sexual consent.

### Decisions to Intervene or Not to Intervene

This theme focuses on what influences bystanders to intervene (or not) in a sexual assault. The areas included are variability in identifying the need for bystander intervention; ambiguity of the event; and peer support.

#### Variability in Identifying the Need to Intervene

Identifying a need to intervene within a sexual assault was a key step identified by the participants. Students in both Australia and the United Kingdom identified similar signs of an impending sexual assault. However, emphasis on when something was wrong was different. Participants in Australia tended to describe a clear situation.

it’s usually body language. [. . .] You can see, if it’s something light, as in your passing by someone, and they slap your butt. And you react and complain and the bystander will look at this, [. . .] body language is the more obvious signal or sign of the abuse. And it draws attention and bystanders can come and see what’s happening. Thomas (M), Australia

A victim would be seen as clearly refusing the perpetrator’s actions, trying to get away, and not giving consent. Some of the Australian participants felt that body language would be the key factor influencing intervening behavior; if it was clear the victim was uncomfortable, the likelihood of bystander intervention increased.

Participants in the UK, on the other hand, focused more on ambiguous situations and the factors that might alert them that something was not right, requiring intervention.

I think the victim would be not as obvious about wanting to get away and stuff because they would be a lot more vulnerable and weaker. Yeah just um, you’re a lot slower when you’re drunk, so if you’re trying to push them away or something. [. . .] I think if they just not, not speaking much, I think they’d really be, um, and struggling to hold themselves up. Emily (F), UK

In an ambiguous situation, alcohol and how the victim is affected by it could be the factor a potential bystander identifies. More broadly, a potential victim’s ability to consent may be diminished. There would be clear signs that the victim is severely intoxicated, such as slowed motor movements, not speaking as much, and struggling to hold themselves up. These would all be noticeable indicators that something is not right and requires intervention. However, the cues between being intoxicated and at risk of sexual assault must be distinguishable from being intoxicated and not at risk of sexual assault.

#### Ambiguity of Event Negatively Impacts Bystander Intervention

The type of situation a bystander is in or encounters when witnessing a sexual assault can strongly impact the decision to intervene or not. If a situation clearly depicts a sexual assault, the likelihood of intervening increases. However, sexual assaults tend to be more ambiguous due to the number of variables present. Bystanders may see an assault, but due to the ambiguity in their assessment of the situation, they may hesitate on intervening.

not sure of the situation, that that’s what’s happening, that it’s sexual assault or like not being aware of what sexual assault is. Thinking that if they don’t know the people that, if they don’t know that’s how they normally act or if that’s what always happens. Maybe they’re a couple, or something. If it’s more. Charlotte (F), Australia

Possible misunderstanding of what sexual assault entails, the lack of clarity of the situation, and the ambiguity around the relationship are some of the possible barriers that could prevent bystander intervention. These barriers generate fear of getting it wrong among potential bystanders. Participant responses supported research by Bennett et al. (2014) in that some bystanders, depending on the situation, may assume something other than an assault is going on, may engage in diffusion of responsibility, or feel they do not have the skills or confidence to intervene.

Somebody who’s not very confident is not going to be very forthcoming about stepping into a situation. They would be very wary about that. Whether it be lacking confidence physically, because it might be somebody 3 times their size. Or confidence in just talking to people. Georgia (F), Australia

The bystander needs to be really good people to stop that. I think it’s easier to omit yourself in this case. Sometimes people just don’t want to actually break their relationships. Thomas (M), Australia

Confidence in one’s ability to intervene could be a major factor affecting bystander intervention, which is in line with past research (Burn, 2009; Exner & Cummings, 2011). Ability to speak outside of their social circle, fear for personal safety, or that the perpetrator may be part of their friendship group could also negatively impact bystander intervention (Humphreys, 2007).

Environmental factors also contribute to the ambiguity of the situation and likelihood of bystander intervention.

it depends on your environment I think [. . .] at a house party or night club you’re not really focused on that, you’re kind of focused on, like why you’re there, which is to have fun and drink and stuff. Lauren (F), UK

The complexity of where sexual assaults take place could increase the difficulty in spotting the signs. If people are there to have fun, they may be less aware of their surroundings and more focused on what they are doing. Bystanders might be under the influence of alcohol themselves, which would then likely impair reaction time and decision-making (Monks et al., 2010), consequently impacting likelihood of intervention.

#### Support Positively Influences Bystander Intervention

There was a difference in opinion regarding whether being surrounded by friends while witnessing a sexual assault would support bystander intervention. Some participants argued that being with friends would provide a distraction, increasing the risk of not intervening.

When they are with friends immediately, they have this sense that other people are around and if something needs doing, then someone will do it, and they don’t actually have to take responsibility. Stephen (M), UK

However, most felt that “if you are on your own, you’re crippled by the fear” (Sophie [F], UK). Instead, having support would facilitate intervening by increasing confidence, enhancing personal safety, and encouraging direct intervention.

I think there’s power in numbers, [. . .] the more people that are empowered with knowledge and tools and methods and skills. [. . .] Them all being able to look at each other and share a social connection, they share a common goal, um, and then I think there is a power there for people to speak together Rebecca (F), Australia

If your friends are with you, you have the confidence anyway. You’re given that boost of you’re in a group, so even if it’s not what you think it is, because [. . .] you are a bit more confident to say something whereas if you are on your own, you’re crippled by the fear [. . .] I think that togetherness is what gives people the confidence to step in when things are you know going wrong Sophie (F), UK

I guess there’s safety in numbers. Like um, if you go by yourself something could happen to you and you don’t want to risk it. So it’s better to not intervene right there and then [. . .] but just maybe report it yourself [. . .] I would feel safer in a group, [. . .] then I could be like we need to intervene here, but I wouldn’t feel comfortable doing it just by myself. Christopher (M), UK

Strength in numbers and being surrounded by people with shared values and beliefs could foster a connection between bystanders, enhancing confidence and facilitating intervention.

## Discussion

This study examined university students’ perceptions and understanding of sexual assault and bystander intervention, in the United Kingdom and Australia. To our knowledge, it is the first study of its kind to qualitatively explore perceptions of bystander intervention. The study provides a more in-depth and nuanced insight of students’ understanding of sexual assault, consent, and bystander intervention. Two research questions were explored, the first of which focused on how university students understand and perceive sexual assault and consent. Participants viewed the definition of sexual assault as ambiguous and agreed that a lack of consent was the key defining factor.

There are different methods of providing consent in a sexual relationship ranging from nonverbal to verbal. However, regardless of method, consent is not a blanket agreement; it is ongoing and continuous and can be revoked at any stage (Shumlick & Fisher, 2020). Within the present study, participants discussed active consent, which they indicated is displayed via nonverbal willingness to engage in sexual activity and the absence of a no. This aligns with Muehlenhard et al.’s (2016) concept of affirmative consent. It has been argued that verbal consent can remove ambiguity regarding someone’s consent to sexual activity (Humphreys, 2007), but can be perceived as awkward and unrealistic (Curtis & Burnett, 2017). However, in our study, participants indicated that nonverbal methods were effective at relaying consent. Furthermore, they suggested that nonverbal methods are obvious and easily interpreted, supporting findings by Beres (2010) and Lewis et al. (2022). Arguably, if this is the case, intervention programs should focus on the more subtle nonverbal behaviors to help bystanders identify whether or not consent is present, as opposed to relying solely on verbal utterances.

The second research question explored university students’ perceptions and understanding of what it means to be a bystander and the factors that influence bystander intervention. Participant responses largely reflected what is present within the USA-based studies. Participants agreed that the first step to bystander intervention was identifying a need to intervene, which is the first step in the bystander model (Latané & Darley, 1970). The cues identified by participants rely on body language. However, Australian participants reported relying on obvious cues of nonconsent, whereas UK participants relied on more subtle cues such as the effects of alcohol and how that removes the ability to consent. The ability to interpret these cues increases the likelihood of bystander intervention (Carlson, 2008; Koelsch et al., 2012; McMahon et al., 2015).

Situational cues affect bystander intervention. Participants in our study said they were more likely to intervene if the situation was clear, which supports research showing that if the seriousness of the situation is clear, the likelihood of intervening increases (Carlson, 2008; Koelsch et al., 2012; McMahon et al., 2015). However, barriers to a clear understanding of the situation decrease the likelihood of bystander intervention (Kania & Cale, 2018). Participants explained that if they were not sure whether there was a lack of consent, or did not know the people well, this would reduce their likelihood of intervening. Other barriers include misjudging the impact of alcohol (Drouin et al., 2018), especially if the victim is not a close friend of the bystander. In party environments, participants reported being more focused on having fun and so were less aware of their surroundings (Burn, 2009), which could cause them to miss signs that something is wrong. Bystanders might be under the influence of alcohol themselves, which could reduce their ability to identify risk (Ham et al., 2019), impair their reaction time and decision-making (Monks et al., 2010; Leone et al., 2018), and reduce the responsibility they feel to intervene (Jozkowski et al., 2021). Alcohol intoxication could also influence bystanders’ interpretations cues (Leone et al., 2018), for example by blaming the victim more and the perpetrator less (Jozkowski et al., 2021). The fear of misinterpreting the situation itself, fear for personal safety, and not wanting to get it wrong can also reduce the likelihood of bystander intervention (Humphreys, 2007). Thus, more education on how to identify signs of a possible sexual assault and how to safely intervene needs to be implemented on university campuses.

Positive peer support was reported to counteract some of these barriers and facilitate intervening, which is in line with prior research (Banyard et al., 2018; Brown & Messman-Moore, 2010). This finding was present among both the female amd male participants, which contradicts past research that demonstrates men may be more fearful of intervening due to the negative perceptions associated with intervening (e.g., Oesterle et al., 2018). Although the present study did not explore this further, this could be due to the sexual assault media cases at the time, as well as the sexual assault awareness campaigns on the university campuses (e.g., Consent is Sexy). Having positive peer support can increase a bystander’s confidence, aid in maintaining personal safety, and encourage direct intervention. This shared interest can positively influence intervening behavior as the bystanders are working toward a common goal. This is representative of the social identity approach, which finds that the group a person associates with often has commonalities and shared beliefs (Hogg & Abrams, 1988; Levine et al., 2005; Tajfel et al., 1971; Tajfel & Turner, 1979). Thus, situating bystander behavior within a social identity approach is important both in research and when designing interventions.

### Limitations and Strengths

The study is based on a purposive sample based across a university in the United Kingdom and in Australia. While the findings are not generalizable to all university students across the United Kingdom and Australia, it is to the authors’ knowledge the first known study to compare findings across two countries in a way that provides valuable insight into student perceptions regarding sexual assault, consent, and bystander intervention. Furthermore, the findings from the current study need to be interpreted within the context of the research design. First, when recruiting participants, every attempt was made to obtain a diverse sample. However, more females were recruited compared to males, although there do not appear to be any gender differences in the responses. Second, the offer of a small monetary reward for students in Australia might have influenced how they participated. Third, at the time of interviews, sexual assault awareness campaigns were being implemented (e.g., Consent is Sexy) on the Australian university campus, which could have influenced participants’ perceptions about consent and bystander intervention.

### Implications and Conclusion

These findings have important implications for researchers and practitioners. The findings demonstrate that a person’s understanding and interpretation of sexual assault and consent is subjective, and consequently, this can impact on bystander intervention. The ambiguity around sexual assault is a major factor limiting when and how people intervene. There are many challenges around sexual consent (Fernet et al., 2021). Our findings show that people are not going to rely solely on explicit verbal consent, which makes it difficult for bystanders to interpret consent clearly. This means that bystander intervention is likely to be a more effective prevention strategy for cases where there the lack of consent is clear and unambiguous, which is likely to be a minority of cases.

There is potential to reduce ambiguities around consent by moving toward affirmative consent (Beres, 2018; Dougherty, 2015), but this is likely to be a long-term aspiration. Increasing education and awareness around sexual assault to enhance understanding and improve clarity around the situations requiring bystander intervention, while acknowledging the complex nature of this topic, could increase the likelihood of intervening and reduce the prevalence of sexual assault (Beres, 2007; Lilley et al., 2023). One way of addressing this is moving toward more experimental, ecologically valid intervention training (e.g., serious games; Labhardt et al., 2017).

Future research should move toward measuring actual helping behavior when witnessing a possible sexual assault, by developing an ecologically valid methodology (Labhardt et al., 2017). In doing so, researchers could explore the complex dynamics involved with bystander intervention such as how intoxication can impact someone’s ability to accurately interpret a situation and how it can affect likelihood of intervening. It can also allow researchers to examine what supports bystanders’ actions, particularly when there are many people present. Future research could further develop our understanding of how peer support might reduce ambiguity, increase confidence, and promote bystander intervention.
